# Diethyl fumarate exhibits potent activity against differentially culturable *Mycobacterium tuberculosis* persisters

**DOI:** 10.3389/fcimb.2026.1901638

**Published:** 2026-10-08

**Authors:** Amelie J. Herbert, Obolbek Turapov, Fraser MacDonald, Irawaty Djaharuddin, Muhammad Nasrum Massi, Douglas B. Kell, Vadim A. Makarov, Galina V. Mukamolova

**Affiliations:** 1 Leicester Tuberculosis Research Group, Infection and Microbiology Division, School of Biological and Biomedical Sciences, University of Leicester, Leicester, United Kingdom; 2 Department of Pulmonology and Respiratory Medicine, Faculty of Medicine, Hasanuddin University, Makassar, Indonesia; 3 Dr. Wahidin Sudirohusodo Hospital, Makassar, Indonesia; 4 Department of Biochemistry, Cell and Systems Biology, Institute of Systems, Molecular and Integrative Biology, Faculty of Health and Life Sciences, University of Liverpool, Liverpool, United Kingdom; 5 Research Center of Biotechnology, Russian Academy of Sciences, Moscow, Russia

**Keywords:** diethyl fumarate, differentially culturable bacteria, dimethyl fumarate, *Mycobacterium abscessus*, persisters, tuberculosis, *Mycobacterium tuberculosis*

## Abstract

Tuberculosis (TB), caused by *Mycobacterium tuberculosis* (Mtb), remains the leading cause of death from a single infectious agent. During infection, Mtb forms drug-tolerant persisters, including differentially culturable (DC) cells that fail to grow on solid media and contribute to prolonged TB treatment and disease relapse. DC Mtb formation is driven by host inflammatory responses, particularly nitric oxide (NO), suggesting that anti-inflammatory agents may limit persister generation. Here, we developed a workflow to evaluate the activity of drug candidates against DC Mtb persisters. This approach was validated using dimethyl fumarate (DMF) derivatives, as DMF has previously been shown to eradicate DC Mtb but is limited by instability and poor solubility. We assessed DMF and nine DMF derivatives for antimycobacterial activity. The workflow included antimicrobial susceptibility testing against *Mycobacterium tuberculosis* (Mtb) and *Mycobacterium abscessus* (Mabs), followed by cytotoxicity evaluation and measurement of NO release in lipopolysaccharide (LPS)-stimulated macrophages. Selected DMF derivatives were further tested against DC Mtb persisters generated *in vitro* using a NO donor as well as persisters recovered from interferon γ-stimulated macrophages. DMF and diethyl fumarate (DEF) inhibited Mtb growth, while ethyl-3-benzoylacrylate (EBL) and methyl-trans-4-oxo-2-pentenoate (MTOP) exhibited bactericidal activity against both Mtb and Mabs. DEF effectively eliminated DC Mtb in infected macrophages, with activity comparable to DMF. This anti-DC Mtb persister effect was associated with reduced NO production, highlighting the importance of anti-inflammatory activity in targeting DC Mtb persisters. DMF and its more soluble and stable derivative DEF represent promising candidates for the development of therapies targeting DC Mtb persisters. Their dual anti-inflammatory and anti-mycobacterial properties support their potential as a novel therapeutic strategy against persistent tuberculosis infections.

## Introduction

Tuberculosis (TB) remains a major global health threat, responsible for an estimated 1.23 million deaths in 2024 (Who, 2025). TB is caused by the obligate pathogen *Mycobacterium tuberculosis* (Mtb), a slow-growing bacterium equipped with a sophisticated arsenal of virulence factors that enable its replication and survival within host macrophages. Current TB treatment typically requires six months and involves a combination of four drugs that target both actively replicating and non-replicating Mtb (Dartois and Rubin, 2022). Shortening treatment duration increases the risk of TB relapse, which, even following treatment completion, has been reported to range from 1.8% (Rosser et al., 2018b) to 11% (Cudahy et al., 2020).

Although the precise bacterial population responsible for relapse is still ambiguous, Mtb is known to generate physiologically diverse subpopulations during infection (Chung et al., 2022; Ajitkumar et al, 2026). These subpopulations differ in growth requirements, metabolic state, lipid composition, and other characteristics. As with other bacteria, non-replicating Mtb are generally believed to exhibit greater tolerance to antimicrobial treatment and are therefore more difficult to eradicate. The term ‘persister’ is used to refer to phenotypic subpopulations of organisms that tolerate concentrations of an antibiotic that would normally kill them (Salcedo-Sora and Kell, 2020). These mycobacteria are thought to adopt a specialised persister-like state and are commonly referred to as Mtb persisters (Zhang et al., 2012).

One clinically relevant subpopulation of Mtb persisters comprises differentially culturable Mtb (DC Mtb), which arise during infection and are highly abundant in clinical samples. DC Mtb lose the ability to form colonies on solid media and require recovery in liquid media, often supplemented with sterile supernatant (CSN) from actively growing Mtb cultures (Mukamolova et al., 2010; Turapov et al., 2016; Chengalroyen et al., 2016; Rosser et al., 2018a; Gordhan et al., 2024), or recombinant resuscitation-promoting factor (Rpf) (Mukamolova et al., 2010; Huang et al., 2014). DC Mtb display increased tolerance to first-line anti-TB drugs (Mukamolova et al., 2010; Turapov et al., 2016) (Mcaulay et al., 2018; Zainabadi et al., 2021), and are associated with unfavourable treatment outcomes (Peters et al., 2022; Decker et al., 2025). In the literature, these bacteria are also referred to as non-culturable (Shleeva et al., 2002) or differentially detectable Mtb (Saito et al., 2017). The term ‘dormancy’ is also widely used to describe such phenotypically non-growing yet resuscitable forms (Kaprelyants et al., 1993).

*Mycobacterium abscessus* (Mabs), an opportunistic environmental pathogen associated with respiratory infections, is also known to form persister subpopulations. It is highly prevalent in patients with cystic fibrosis, other risk factors include pre-existing pulmonary disease, bronchiectasis and history of mycobacterial disease (Victoria et al., 2021). Furthermore, the highly intrinsically resistant nature of this organism contributes to low treatment success rates of 30-50% (Victoria et al., 2021).

Host inflammatory factors have been proposed as key triggers for DC Mtb formation *in vivo* (Turapov et al., 2014), including nitric oxide (NO) (Gap-Gaupool et al., 2024) and reactive oxygen species (Saito et al., 2021). Accordingly, several laboratory models have been developed to generate DC Mtb both *in vitro* (Saito et al., 2017; Gap-Gaupool et al., 2024), and *in vivo* (Hu et al., 2015; Glenn et al., 2022). Recent studies suggest that anti-inflammatory compounds can enhance the eradication of Mtb persisters and DC Mtb (Glenn et al., 2022). We therefore hypothesised that drugs with combined anti-inflammatory and anti-Mtb activity may represent an effective strategy for targeting Mtb persisters.

We have previously shown that the approved anti-inflammatory drug dimethyl fumarate (DMF) eliminated DC Mtb in infected animals (Glenn et al., 2022). However, the precise mechanism of action of DMF remains under debate. DMF is rapidly converted *in vivo* into monomethyl fumarate (Bruck et al., 2018), suggesting that it acts as a prodrug. DMF is unstable at room temperature and tends to decompose during storage under moist conditions. Furthermore, DMF has poor solubility and requires high doses to be effective in animal models (Glenn et al., 2022).

In this study, we evaluated several commercially available DMF derivatives (molecules with structural “similarity” to DMF, **Figure 1**) to improve our understanding of their effects on Mtb persisters and to guide future drug optimisation, while also evaluating the potential for these compounds to combat non-tuberculous mycobacteria, primarily Mabs. Following an initial assessment of antimicrobial activity and cytotoxicity, selected compounds were tested against DC Mtb generated *in vitro* using exposure to a sustained NO donor and in infected murine macrophages stimulated with interferon γ (IFN-γ). Our results demonstrate that DMF has no significant effect on DC Mtb generated in the NO-donor model but effectively eliminates DC Mtb in infected macrophages. Monomethyl fumarate (MMF) displayed reduced growth inhibitory activity against Mtb, whereas diethyl fumarate (DEF) was more potent, exhibiting a lower minimum inhibitory concentration and an effect on DC Mtb in macrophages comparable to that of DMF. In contrast, ethyl-3-benzoylacrylate (EBL) and methyl-trans-4-oxo-2-pentenoate (MTOP) showed no significant activity against DC Mtb persisters but were bactericidal against actively growing Mtb and Mabs. Overall, this work establishes a solid basis for the further modification of DMF-based compounds aimed at developing more potent therapies targeting Mtb persisters.

**Figure 1:**
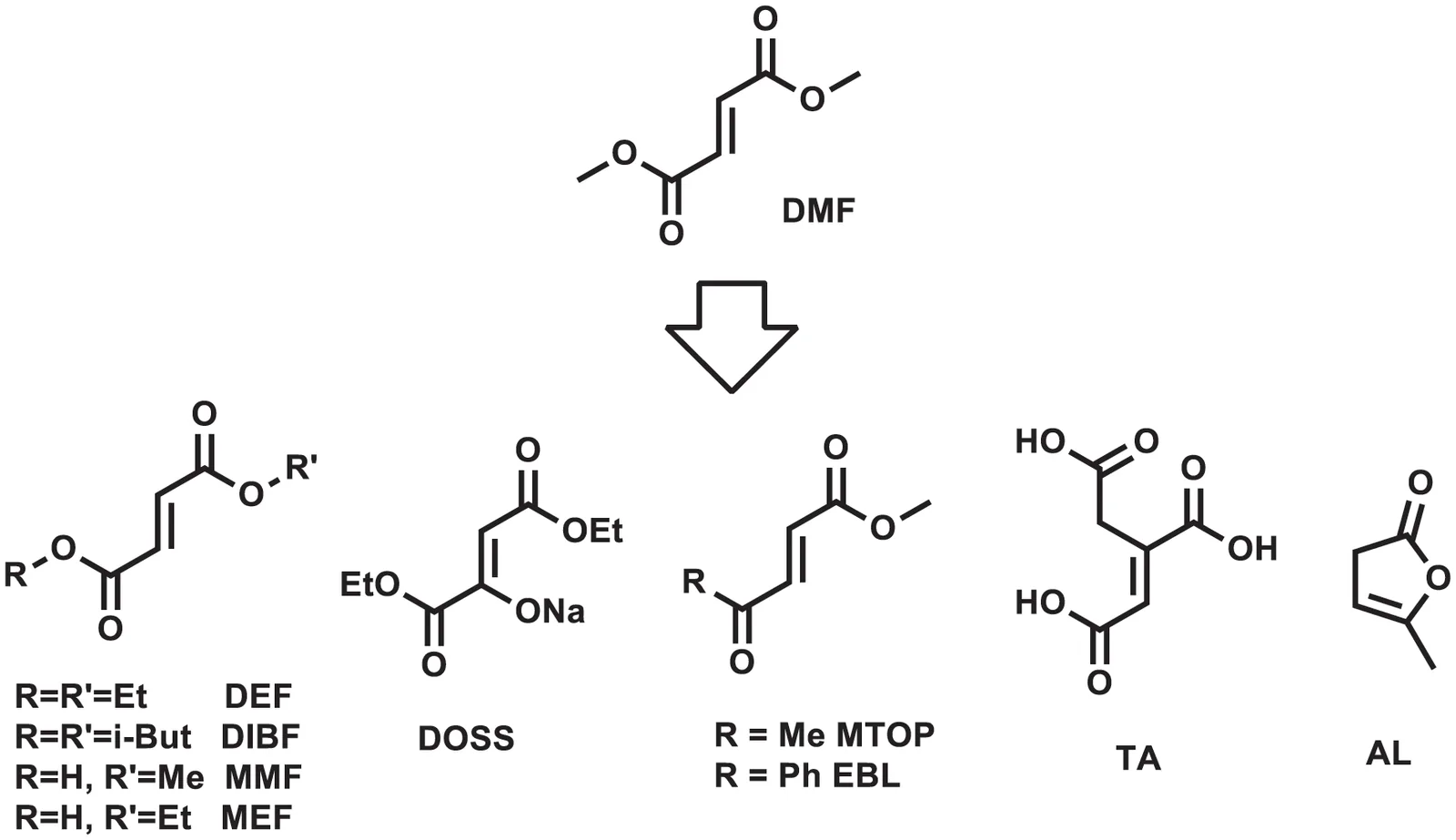
Chemical structures of selected DMF derivatives. The medicinal chemistry rationale underlying the selection of these derivatives was to balance structural similarity to DMF with sufficient diversity. This approach enables identification of the essential and non-essential atoms and molecular fragments responsible for biological activity.

## Materials and methods

### Organisms and media

*Mycobacterium tuberculosis* H37Rv (Mtb), obtained from Professor W.R. Jacobs (Albert Einstein College of Medicine) and *Mycobacterium abscessus* ATCC19977 (Mabs, smooth and rough morphotypes) were used for testing drug candidates. Mycobacteria were routinely cultured in liquid Middlebrook 7H9 broth supplemented with 0.2% (v/v) glycerol, 10% (v/v) ADC (albumin-dextrose complex) or OADC (oleic acid albumin dextrose catalase, Becton, Dickinson and Company) and 0.05% Tween 80 (hereafter referred to as 7H9 medium). For solid culture, Middlebrook 7H10 agar was supplemented with 10% (v/v) ADC or OADC, in selected experiments charcoal was added at a final concentration of 0.2% (w/v). For each experiment, bacteria were grown from frozen stocks in 7H9 medium at 37 °C with shaking at 100 rpm to mid-logarithmic phase, corresponding to an optical density at 580 nm (OD_580_) of 0.5-0.8. Cultures were then reinoculated into fresh medium and grown to an OD_580_ of 0.5-0.8. These second-passage cultures were used immediately after OD_580_ measurement for all subsequent experiments. At least three independent cultures initiated from frozen stocks were used as biological replicates.

Mabs strains exhibited stable smooth and rough morphotypes. Unless otherwise stated, most experiments were performed using the smooth morphotype because it was less prone to aggregation.

The murine macrophage cell line J774A.1 was maintained in Dulbecco’s modified Eagle’s medium (DMEM) supplemented with 10% (v/v) foetal bovine serum (FBS) at 37 °C with 5% CO_2_. The human monocytic cell line THP1 was cultured in RPMI-1640 supplemented with 10% (v/v) FBS at 37 °C with 5% CO_2_.

Dimethyl fumarate and related compounds were obtained from Sigma-Aldrich and were used without additional purification; a complete list of compounds and stock preparation details are provided in **Table 1**. All stock solutions were freshly prepared in DMSO and used on the day of the experiment. DMSO alone was used as a control. We noted that DMF tended to lose its activity over time during storage.

**Table 1:** Summary of tested compounds including MIC values.

Compound	CAS	Abbreviation	Stock, mM	MIC Mtb, µM*	MIC Mab smooth morphotype, µM*
Dimethyl fumarate	624-49-7	DMF	200	25-100	>1000
Diethyl fumarate	623-91-6	DEF	600	12.5	>1000
Di-isobutyl fumarate	7283-69-4	DIBF	400	1500	>1000
Diethyl oxalacetate sodium salt	40876-98-0	DOSS	200	1500	>1000
Mono-methyl fumarate	2756-87-8	MMF	700	200	>1000
Mono-ethyl fumarate	2459-05-4	MEF	700	200	>1000
Methyl-trans-4-oxo-2-pentenoate	2833-24-1	MTOP	700	3.13	50
Ethyl-3-benzoylacrylate	17450-56-5	EBL	700	3.13	50
Trans-Aconitic acid	4023-65-8	TA	500	3000	>1000
Angelica lactone	591-12-8	AL	1000	>3000	>1000
Ibuprofen	15687-27-1	IBU	400	500-1000	>1000
Sodium diclofenac	15307-79-6	DIF	300	400	>1000
Dexamethasone	50-02-2	DEX	300	750	>1000
Rifampicin	13292-46-1	RIF	10	0.1	6.25
Streptomycin	57-92-1	STM	170	1.35	>100
Amikacin	37517-28-5	AMK	85	N/D	3.13

*Data represent three independent experiments performed in technical triplicate. MICs were determined from OD_580_ measurements.

### Determination of antimicrobial susceptibility

Minimum inhibitory concentrations (MICs) were determined using a broth microdilution assay. Compounds were serially diluted two-fold in 96-well plates containing 7H9 medium. Drug-free and bacteria-free controls were included in each experiment. At least three technical replicates were prepared for each condition. Each well was inoculated with 1-5x10^5^ bacteria from second passage cultures. Plates were sealed with polyvinyl tape and incubated statically at 37 °C for 1 week (Mabs) or 2 weeks (Mtb). No additional oxygenation was provided, and bacteria exhibited normal growth as assessed by OD_580_ measurement and further confirmed by colony-forming unit (CFU) enumeration and microscopy.

Bacterial growth was assessed by measuring OD_580_ using Varioskan plate reader (Thermo Fisher Scientific). In some experiments resazurin was added to visualise cell growth to a final concentration of 0.05% (w/v) and incubated for an additional 24 h, followed by fluorescence measurement (excitation 540 nm, emission 590 nm) using the Varioskan plate reader (Thermo Fisher Scientific). MICs were defined as the lowest drug concentrations at which no detectable growth was observed based on absorbance and fluorescence readings.

To assess the effects of compounds on mycobacterial viability, Mtb or Mabs cultures were incubated with selected compounds in 7H9 at 37 °C with shaking at 100 rpm for up to 10 days. Untreated controls were included in all experiments. Prior to determination of colony forming unit (CFU) counts, 1 mL aliquots were centrifuged at 3,000 x g for 20 min, and the bacterial pellets were resuspended in fresh 7H9 medium. For Mtb experiments, DMF was used at concentration of 100 μM, DEF, MTOP and EBL were used at 25 μM. For Mabs experiments, MTOP and EBL were used at a concentration of 250 μM. At least three biological replicates were used.

### Cytotoxicity assays

Two complementary methods were used to assess the effects of drugs on macrophage viability: (i) the 3-[5-dimethylthiazol-2-yl]-2,5 diphenyl tetrazolium bromide (MTT) assay which measures cellular metabolic activity was used with J774A.1 cells; and (ii) the Trypan blue exclusion assay which assesses membrane integrity was used with THP1 cells because these cells do not adhere to plastic and can be counted using a haemocytometer without requiring additional detachment procedures. Triplicate samples were prepared for each condition.

For the MTT assay, a commercial MTT Cell Growth Assay Kit (Millipore) was used according to the manufacturer’s instructions. Briefly, 1x10^4^ J774A.1 cells per well were seeded into 96-well plates containing DMEM. Test compounds were added at concentrations ranging from 1 μM to 200 μM, and plates were incubated statically for 24 h at 37 °C in a humidified incubator with 5% CO_2_. MTT reagent was added to each well to a final concentration of 0.5 mM and incubated for 4 h at 37 °C, followed by the addition of an equal volume of 10% (w/v) SDS in 10 mM HCl solution and further incubation for 4 h. Absorbance was measured at 580 nm and background values were subtracted prior to analysis. Cell viability was calculated as: Viability (%) = (Abs_580_treated cells/Abs_580_control cells) x 100.

The Trypan blue exclusion assay was validated using mixtures containing known proportions of live and dead cells. Dead cells were generated by treatment with 35% (v/v) ethanol for 5 min, washed, stained with Trypan blue, and confirmed as non-viable by microscopy. Dead and live cells were then mixed at defined ratios and counted microscopically. The correlation between expected and observed percentages of live and dead cells exceeded 80%.

THP1 cells were seeded at 1x10^4^ cells per well in 96-well plates containing RPMI 1640. Following incubation with compounds for 24 h at 37 °C with 5% CO_2,_ Trypan blue was added at a final concentration of 0.2% (w/v), gently mixed and incubated for 10 min. Cells were counted using a Neubauer haemocytometer and a Nikon TMS Inverted microscope. Trypan blue-stained cells were considered non-viable. Cell viability was calculated as:

Viability (%) = (Unstained cells/Total cell count) x 100.

In control experiments, J774A.1 cells were grown on sterile coverslips immersed in DMEM in 6-well plates and treated with 25 mM DMF, DEF, MTOP, or EBL for 24 h. Control cells were treated with DMSO only. Following treatment, coverslips were stained with Trypan blue and examined by microscopy. Cells were counted in 6–10 separate fields of view for each coverslip.

### Determination of compound effects on nitric oxide release

J774A.1 cells (2.5 × 10^5^ cells/mL) were seeded in 48-well plates containing 0.5 mL DMEM. After 24-h incubation at 37 °C with 5% CO_2,_ the culture medium was replaced with DMEM containing lipopolysaccharide (LPS, Thermo Fisher Scientific) at 1 µg/mL. Test compounds were added at specified concentrations, followed by incubation for an additional 24 h at 37 °C with 5% CO_2_.

Nitrite, a stable product of NO oxidation, was measured using a Griess Reagent System (Promega, UK) according to the manufacturer’s instructions. Untreated controls, DMSO solvent controls, and no-LPS controls were included. All conditions were tested in triplicate, and experiments were performed twice. For the positive control experiments aminoguanidine (CAS 79-17-4), a known inhibitor of murine inducible nitric oxide synthase (iNOS), was added at 500 μM prior to compound treatment.

### Testing compounds against DC Mtb persisters

Previously developed models were employed to generate DC Mtb (Gap-Gaupool et al., 2024). The nitric oxide donor (NOD) was synthesised by Dr Vadim Makarov as described before (Gap-Gaupool et al., 2024).

For the NOD model, second-passage mid-logarithmic-phase Mtb cultures (OD_580_ = 0.7-0.9), grown in 7H9 medium at 37 °C with shaking at 100 rpm, were inoculated into 30 mL tubes containing 5 mL of 7H9 medium at a final OD_580_ of 0.1. NOD was added to a final concentration of 100 μM, followed by the addition of selected compounds at 25 μM. Cultures were incubated statically with NOD and the compounds for 24 h at 37 °C. Following centrifugation at 3,000 x g for 20 min, bacterial pellets were resuspended in 1 mL 7H9. Bacterial clumps were dispersed using a Luer-lock syringe fitted with a 31-gauge blunt needle (Harvard Apparatus) by passing the suspension through the needle five times. The resulting suspensions were used for CFU and Most Probable Number (MPN) count determination. Controls included untreated Mtb and NOD-treated Mtb receiving DMSO vehicle only.

For the macrophage infection model, 1x10^5^ J774A.1 macrophages were seeded into each well of a 24 well plate containing 1 mL DMEM. Cells were stimulated with interferon γ (IFN-γ) at 10 ng/mL for 24 h prior to infection with Mtb at a multiplicity of infection (MOI) of 1 for 3 h. For infection experiments frozen Mtb stocks in DMEM with previously determined Mtb CFU counts (~1x10^8^ cells/ml) were thawed at room temperature, carefully dispersed using a blunt needle as described above, and diluted in PBS. Extracellular bacteria were eliminated by exposure to amikacin (200 μg/mL) for 1 h, followed by washing and incubation for an additional 24 h. Adherent macrophages were lysed with 0.2% (v/v) Triton X-100, and lysates were immediately used for CFU and MPN count analyses. Four replicates were included for each condition.

### CFU and MPN count determination

The Most Probable Number (MPN) assay, also known as the limiting dilution-to-extinction assay, enables quantification of resuscitable bacteria and distinguishes them from colony-forming bacteria. Comparison of CFU and MPN counts therefore allows detection of differentially culturable (DC) *M. tuberculosis* populations that do not form colonies on solid medium but can resuscitate in liquid medium supplemented with culture supernatant obtained from actively growing *M. tuberculosis* cultures.

For CFU determination, bacterial suspensions were serially diluted in 7H9, and four 10-μL drops from each dilution, including undiluted cultures, were spotted onto 7H10 agar plates. For drug-exposure experiments 100 μL of undiluted culture was spread onto 7H10 agar supplemented with 0.2% (w/v) activated charcoal. When MPN assays were performed, CFU counting was determined directly from MPN dilution series by spotting 10 μL from each dilution onto 7H10 agar plates. Plates were sealed and incubated statically at 37 °C for 1 week (Mabs) or 3–6 weeks (Mtb).

For MPN assays, a separate 48-well plate was used for each sample. Briefly, 50 μL of bacterial suspension was added to wells containing either 450 μL of either 7H9 medium or 7H9 supplemented with culture supernatant (50:50, v/v) referred to as CSN. Preparation of CSN was described previously (Turapov et al., 2016). Briefly, a 1 L roller bottle containing 200 mL of 7H9 medium was inoculated with 1 mL of a second-passage H37Rv *Mtb* culture and incubated at 37 °C on a roller apparatus for 5–6 days until an OD_580_ of 0.6-0.9 was reached. Cultures were centrifuged at 4,000 × *g* for 20 min, and the supernatant was filter-sterilised twice using separate 0.22 μm filter units. The resulting CSN was used immediately or freeze-dried for future applications. Plates were sealed with polyvinyl tape and incubated statically at 37 °C for up to 12 weeks. The MPN calculator programme was used for determination of MPN counts (Jarvis et al., 2010). Resuscitation indices (RIs) were calculated for 7H9 medium and CSN: RI_7H9 = Log_10_ (MPN_7H9 mL^-1^) − Log_10_ (CFU_7H9 mL^-1^) and RI_CSN = Log_10_ (MPN_CSN mL^-1^ − Log_10_ (CFU_CSN mL^-1^).

### Statistical analysis

At least three independent biological replicates were included in each experiment, and all experiments were performed at least twice. Unpaired two-tailed t-test or one-way ANOVA were applied as appropriate. All analyses were performed using GraphPad Prism 11.

## Results

### Initial screening identified dimethyl fumarate derivatives with antimicrobial activity across two mycobacterial species

Several compounds were selected for initial testing against slow-growing *Mycobacterium tuberculosis* (Mtb) and fast-growing *Mycobacterium abscessus* (Mabs). These included nine commercially available derivatives of dimethyl fumarate (DMF) and three drugs with known anti-inflammatory activity representing distinct mechanisms of action while all impacting on the expression of iNOS (**Table 1**; **Figure 1**).

From a medicinal chemistry perspective, we sought to identify the structural features of DMF essential for activity and those amendable to modification to improve pharmacological properties. We therefore examined a panel of compounds with targeted modifications, deletions, or partial retention of key DMF moieties.

The closest structural analogues included compounds in which the methyl ester groups of DMF were replaced with ethoxy or butoxy carbonyl substituents, diethyl fumarate (DEF) and di-isobutyl fumarate (DIBF), or in which one ester group was hydrolysed to yield the corresponding carboxylic acid, monomethyl fumarate (MMF) and monoethyl fumarate (MEF). Two additional compounds, methyl-trans-4-oxo-2-pentenoate (MTOP) and ethyl-3-benzoylacrylate (EBL), are structurally related to DMF but contain a keto group in place of one ester moiety. Diethyl oxalacetate sodium salt (DOSS) is similarly related to DMF; however, replacement of a proton at the double bond with a hydroxyl group substantially alters its physicochemical properties. Trans-Aconitic acid (TA) consists of a demethylated DMF scaffold with an additional carboxyl group. In contrast, angelica lactone (AL) represents a reduced cyclic analogue of DMF, containing only a single ester functionality. Collectively, these nine compounds capture the key structural variations of the DMF scaffold and thus provide a representative chemical diversity for primary investigating structure–activity relationships in this study (**Table 1**).

To assess anti-persister activity, we developed a workflow that included determination of minimum inhibitory concentrations (MICs), assessment of mycobacterial viability and compound cytotoxicity, evaluation of nitric oxide (NO) released by macrophages as a measure of anti-inflammatory activity, and subsequent testing against DC Mtb persisters (**Figure 2**).

**Figure 2:**
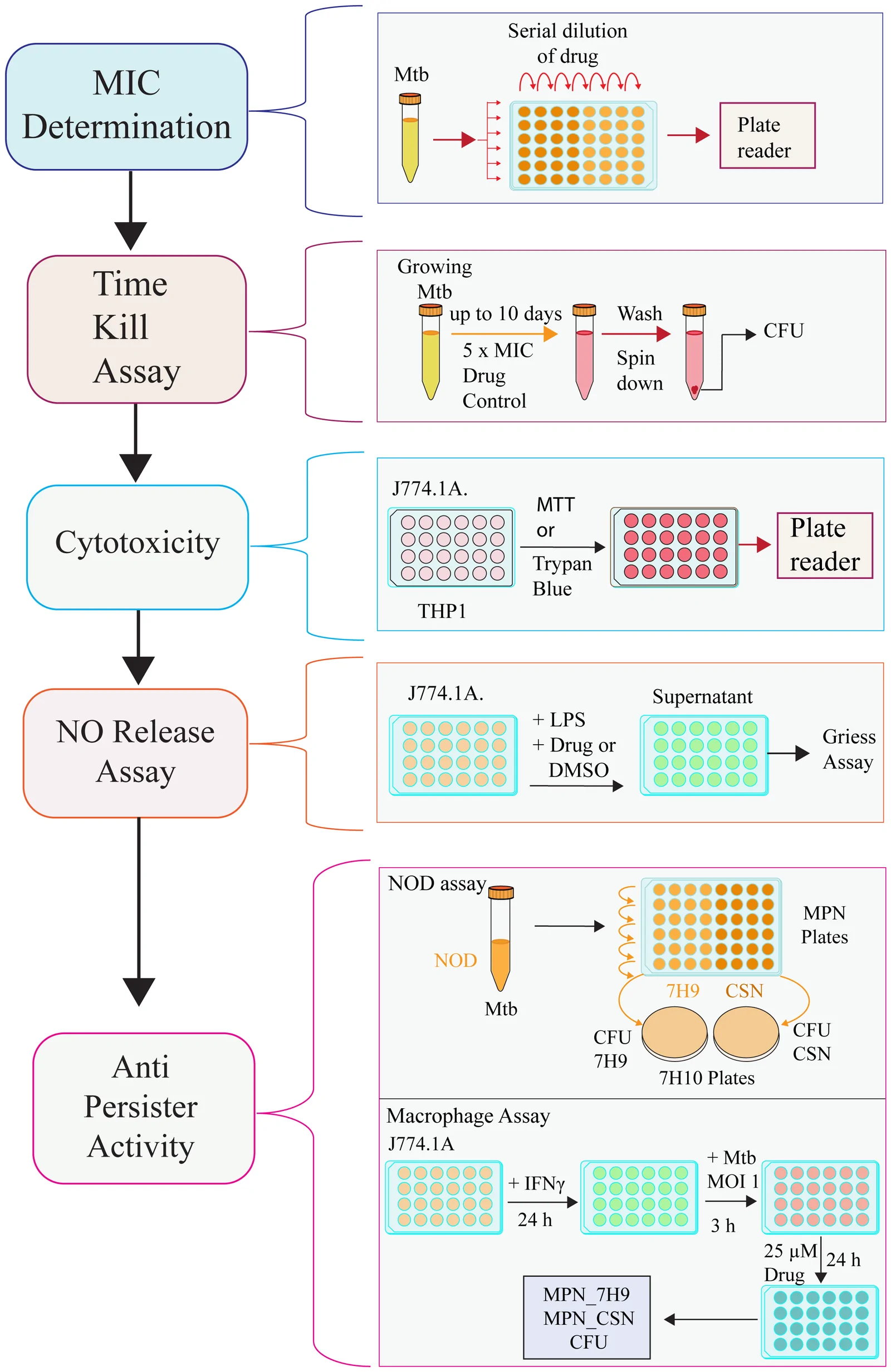
A schematic presentation of workflow for testing compound activity against DC Mtb persisters.

Consistent with previous findings, DMF inhibited the growth of Mtb at 25 µM (Glenn et al., 2022). However, we observed variability in the antimicrobial activity of DMF between batches. Several derivatives, including MMF, a metabolite produced *in vivo* from DMF (Bruck et al., 2018), showed markedly reduced activity with MICs ranging from 200 to 1500 µM. TA and AL were inactive at all concentrations tested.

In contrast, three derivatives exhibited increased activity with lower MIC values: DEF (MIC = 12.5 µM), MTOP (MIC = 3.13 µM) and EBL (MIC = 3.13 µM). Testing against Mabs revealed that only MTOP and EBL inhibited bacterial growth, each with an MIC of 50 µM. Both smooth and rough variants of Mabs were tested and identical MIC values were obtained.

FDA approved anti-inflammatory drugs, ibuprofen, diclofenac and dexamethasone, showed limited inhibitory activity against Mtb and no detectable activity against Mabs (**Table 1**). Therefore, these agents were not investigated further.

Rifampicin and streptomycin were used as positive controls because of their well-established anti-Mtb activity. They exhibited MIC values of 0.1 and 1.35 µM against Mtb, and 6.25 and >100 against Mabs, respectively. Additionally, amikacin was used as a positive control for Mabs and exhibited an MIC of 3.13 µM.

We next tested whether DMF, DEF, MTOP and EBL exerted bactericidal effects against mycobacteria. Mtb cultures were treated with DMF (100 µM), or DEF, MTOP and EBL (25 µM) and incubated at 37 °C with shaking. Bacterial viability was monitored by determining CFU counts over a 10-day period.

As shown in **Figure 3A**, DMF and DEF did not substantially reduce CFU counts in Mtb cultures, indicating predominantly bacteriostatic activity under these conditions. Nevertheless, after 10 days of treatment, CFU counts in DMF-treated cultures were significantly lower than those in the DMSO control, corresponding to an approximately 0.5 log_10_ reduction (*p* < 0.01, unpaired *t*-test). By comparison, DEF treatment did not reduce bacterial viability; CFU counts increased slightly at the start of incubation and remained essentially unchanged thereafter. MTOP treatment resulted in a transient one log_10_ reduction in CFU counts. In contrast, EBL caused a pronounced decrease during the first 7 days, reaching nearly a three-log_10_ reduction, followed by partial recovery by day 10. Notably, both EBL and MTOP displayed bactericidal activity against the smooth variant of Mabs (**Figure 3B**) Treatment with either compound resulted in a rapid decline in CFU counts within the first 24 h and further reduction over 3 days and regrowth by day 7. Considerable variability in CFU counts was observed between biological replicates for both compounds, suggesting sensitivity to experimental variations such as initial bacterial load. DMF and DEF were inactive on Mabs (**Table 1**).

**Figure 3:**
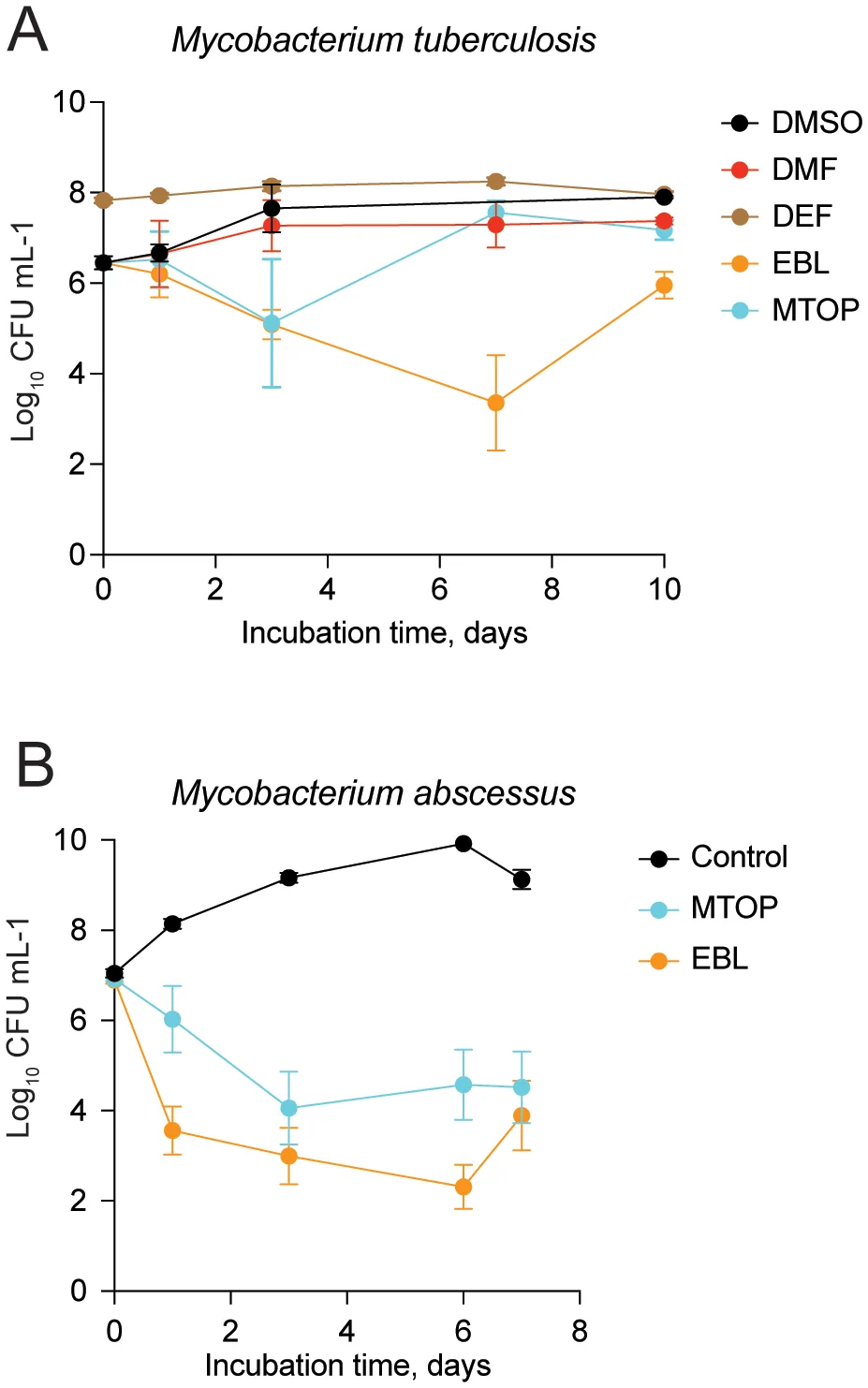
Effect of DMF and its derivatives on the viability of Mtb **(A)** and the smooth morphotype of Mabs **(B)** over time. The limit of detection was log_10_ = 1.38. Two and five independent experiments were performed for Mtb and Mabs respectively, with each experiment conducted in biological triplicate. **(A)** Results from a representative experiment are shown and presented as mean ± SEM (N = 3). **(B)** Data are presented as mean ± SEM (N = 15).

Overall, these results indicate that DMF and DEF primarily exerted bacteriostatic effects against Mtb and were inactive against Mabs, whereas EBL and MTOP displayed bactericidal activity against both mycobacterial species tested.

### DMF and three DMF derivatives display favourable selectivity indices

Next, the effects of the selected compounds on macrophage viability were assessed using two complementary methods: an MTT assay which measures metabolic activity and direct cell viability assessment based on Trypan blue staining following treatment with DMF and its derivatives.

Although the MTT assay showed substantial variability in absorbance values, the overall trends in J774A.1 viability were consistent (**Figure 4A**) and matched the Trypan blue results obtained with THP1 cells (**Figure 4B**). Based on these data, IC_50_ values were calculated for all compounds. DMF and DEF showed similar cytotoxicity, with IC_50_ values of 144 µM and 138 µM, respectively, whereas MTOP and EBL were more cytotoxic, with IC_50_ values of 34 µM and 24 µM (**Figure 4**).

**Figure 4:**
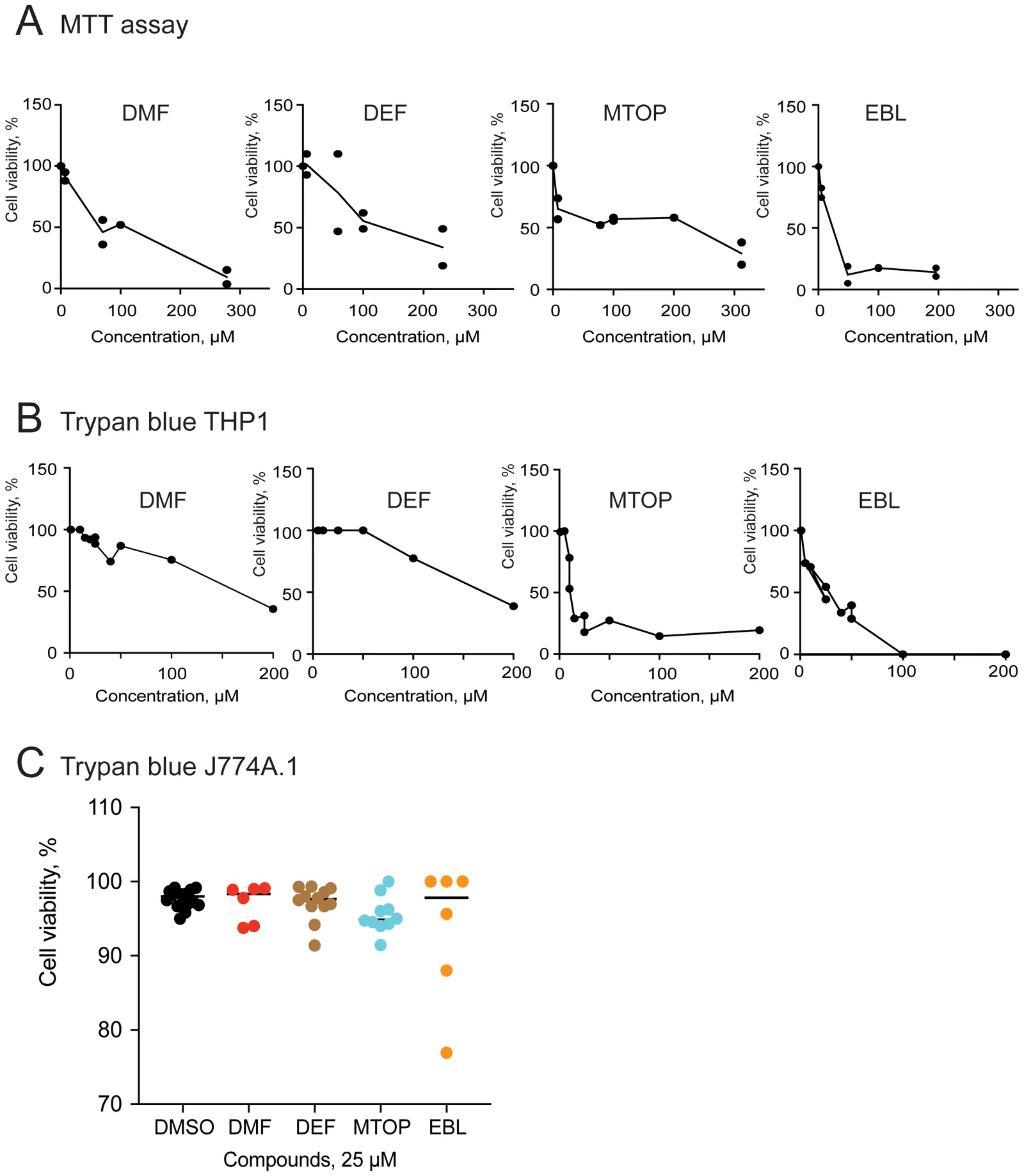
Effect of DMF and its derivatives on macrophage viability. Viability of J774A.1 cells was assessed using the MTT assay **(A)** or Trypan Blue staining **(C)**. Viability of THP1 cell was assessed using Trypan Blue staining **(B)**. **(A)** Cell viability was calculated from MTT absorbance values in treated cells relative to untreated controls, as described in the Methods section. Mean values of technical duplicates from two independent experiments are shown. **(B)** Viability was determined by microscopic examination following Trypan Blue staining, with more than 200 cells counted per condition. IC_50_ values were calculated based on Trypan Blue staining results. **(C)** J774A.1 cells grown on coverslips were treated with 25 μM of each compound or DMSO for 24 h, stained with Trypan Blue and examined microscopically. Values represent counts from individual microscopic fields.

The effects of DMF and its derivatives at 25 µM were further assessed in J774A.1 macrophages by Trypan blue staining (**Figure 4C**). None of the compounds, except EBL, reduced the number of adherent macrophages. EBL induced cell detachment, although the remaining adherent cells remained viable. No significant differences in viability were observed, indicating that all compounds were non-toxic at 25 µM.

MICs, IC_50_ values, and selectivity indices are summarised in **Table 2**. The DMF derivatives displayed higher selectivity indices than DMF, suggesting that these compounds may be suitable for further evaluation as potential anti-inflammatory agents and candidates targeting Mtb persister populations.

**Table 2:** Selectivity indices for DMF and DMF derivatives.

Compound	MIC Mtb, µM	IC50, µM	Selectivity index*
DMF	25-100**	143.7	1.44- 5.75
DEF	12.5	138.1	11
MTOP	3.13	34.1	10.9
EBL	3.13	23.5	7.5

*Selectivity Index= IC50/MIC.

**Includes MIC variation depending on DMF batches

### DMF and DEF suppress nitric oxide release by LPS-stimulated murine macrophages

Nitric oxide (NO), a marker of the inflammatory response, has been shown to induce the formation of DC Mtb persisters both *in vitro* and in infected macrophages (Gap-Gaupool et al., 2024). We therefore assessed the effects of DMF derivatives on NO production using LPS-stimulated J774A.1 macrophages (Noda and Amano, 1998) and quantified nitrite, a stable NO oxidation product, using the Griess assay.

In activated macrophages the nitrite concentration was 26 μM compared with 0.6 μM of nitrite in non-stimulated macrophages. All compounds reduced NO release at 25 μM (**Figure 5**). DMF and DEF were the most potent inhibitors, reducing NO production to 5.5 μM and 3.8 μM, respectively (**Figure 5A**), and further suppressing NO release at 50 μM to 0.8 and 1 μM, respectively. This was also associated with decreased metabolic activity as shown via the MTT assay (**Figure 4A**). EBL and MTOP also reduced NO release, to 12 and 9.5 μM, respectively, although this effect may have been partially attributable to their greater macrophage toxicity. Consequently, the effects of EBL and MTOP at 50 μM were not evaluated.

**Figure 5:**
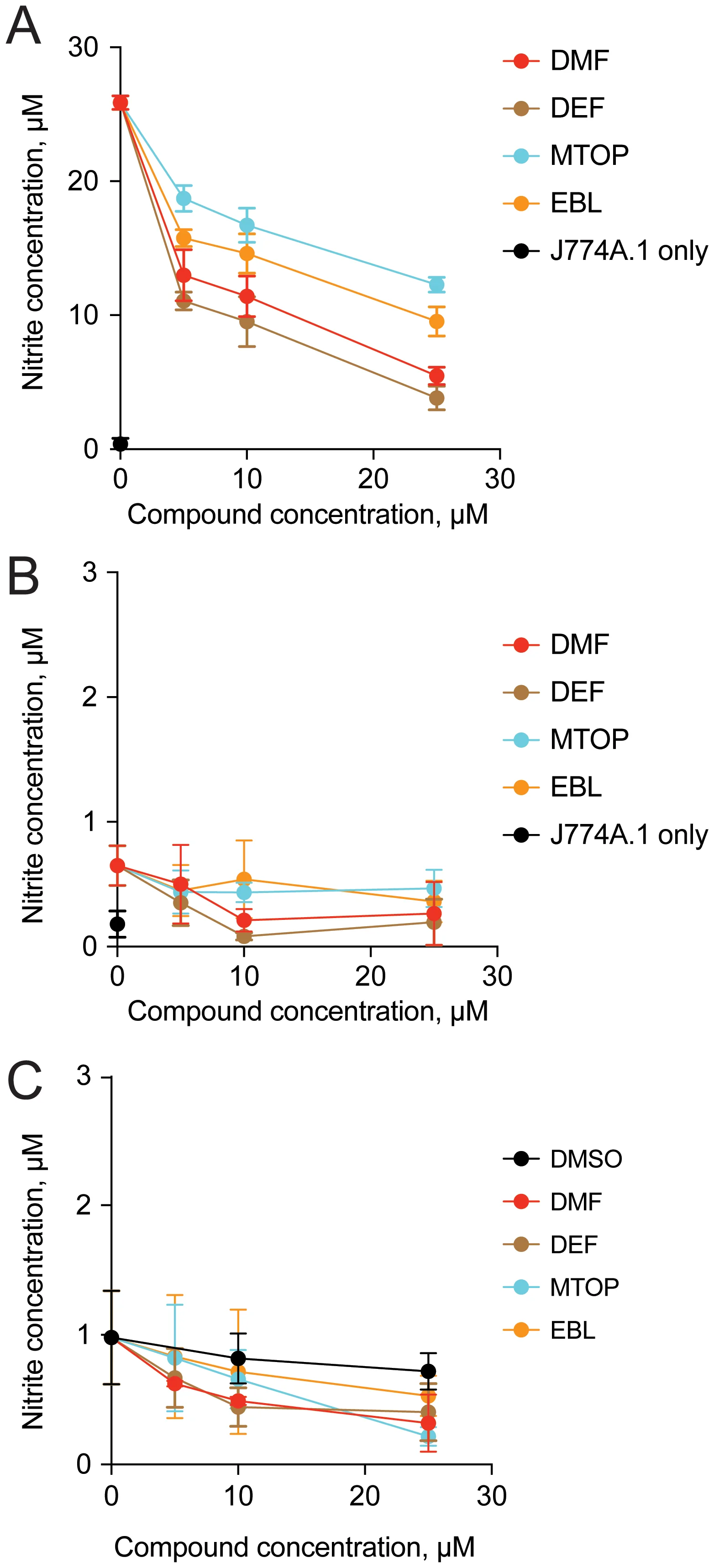
Effect of DMF and DMF derivatives on nitric oxide (NO) release by LPS-stimulated cells **(A, B)** or unstimulated **(C)** J774A.1 macrophages. Nitrite levels were measured using the Griess assay, as described in the Methods. **(A)** J774A.1 cells were stimulated with LPS and treated with DMF or its derivatives. **(B)** Control experiments in which J774A.1 cells were treated with the iNOS inhibitor aminoguanidine (500 μM), stimulated with LPS (1 μg/mL), and exposed to DMF or its derivatives. **(C)** Unstimulated J774A.1 cells were treated with DMSO or DMF derivatives. Two independent experiments were performed. Results from one representative experiment are shown as mean ± SEM (N = 3).

Aminoguanidine, a well-characterised inhibitor of inducible nitric oxide synthase (iNOS), served as a control (**Figure 5B**). Under all conditions, nitrite concentrations remained below 1 μM, confirming that NO production in this system was primarily mediated by iNOS. Unstimulated macrophages produced approximately 1 μM nitrite, and DMSO had no effect (**Figure 5C**). Although the compounds appeared to reduce basal nitrite levels, these differences were not statistically significant (one-way ANOVA, *p* > 0.05).

Together these results demonstrate that DMF and DEF are potent inhibitors of NO release in LPS-stimulated murine macrophages.

### DMF and DEF eliminate DC Mtb persisters in a macrophage infection model

We have previously shown that DMF eliminates DC Mtb persisters in a macrophage infection model (Gap-Gaupool et al., 2024) and infected animals (Glenn et al., 2022). However, it remained unclear whether this effect resulted from a direct action of DMF on DC Mtb or indirectly through modulation of the macrophage environment. To address this question, we employed two complementary models for generation of DC Mtb: (i) an *in vitro* model involving exposure to a custom NO donor (NOD) (**Figure 6**), and (ii) infection of IFN-γ-stimulated J774A.1 macrophages (**Figure 7**).

**Figure 6:**
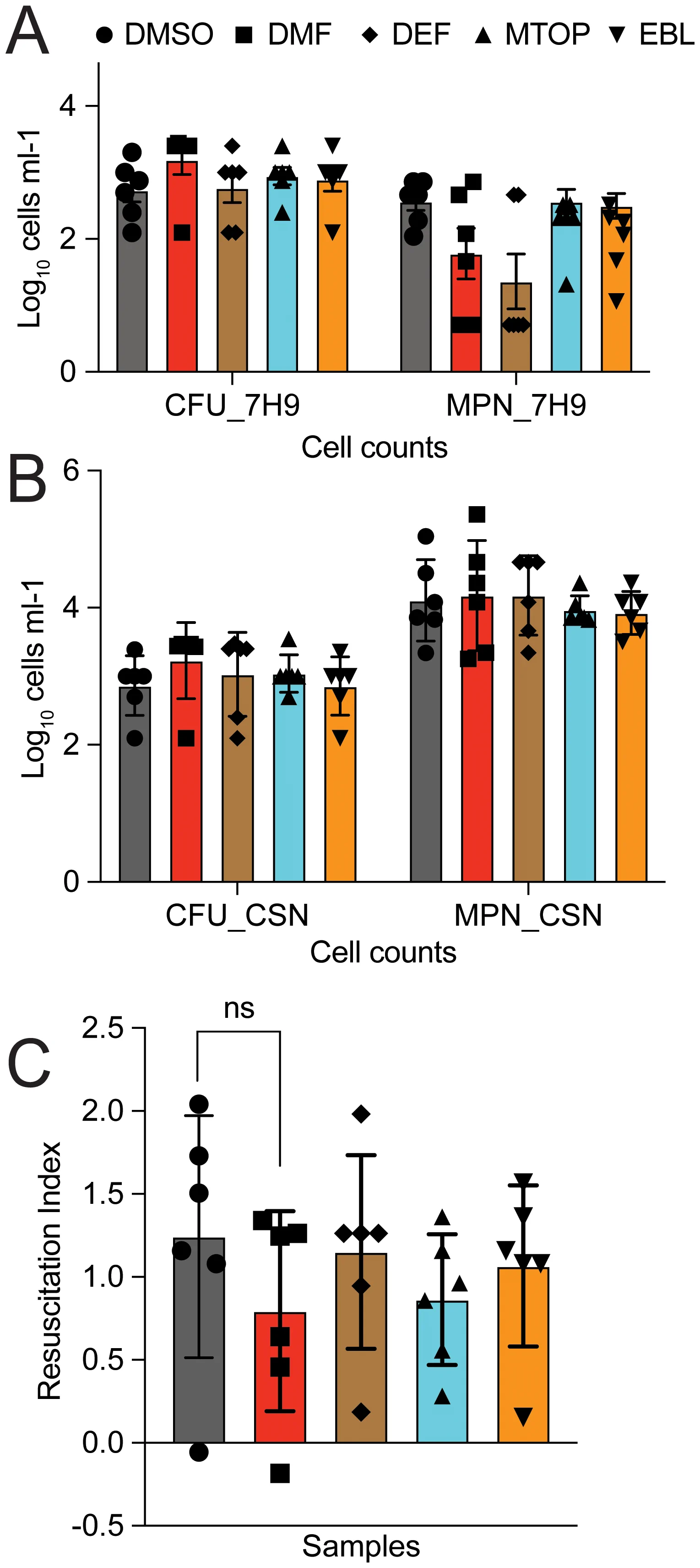
Effect of DMF and DMF derivatives on DC Mtb persisters generated after nitric oxide donor (NOD) exposure. Mtb cultures from logarithmic cultures were treated with NOD (100 μM) and compounds (25 μM) for 24 h prior to determination of CFU and MPN counts. **(A)** CFU_7H9 and MPN_7H9 counts and **(B)** CFU-CSN and MPN_CSN were determined after 24-h exposure. **(C)** The resuscitation index (RI) was calculated as: RI_CSN = log_10_(MPN_CSN mL^-1^) − log_10_(CFU_CSN mL^-1^). Results from two independent experiments performed in biological triplicate are shown as mean ± SEM (N = 6).

**Figure 7:**
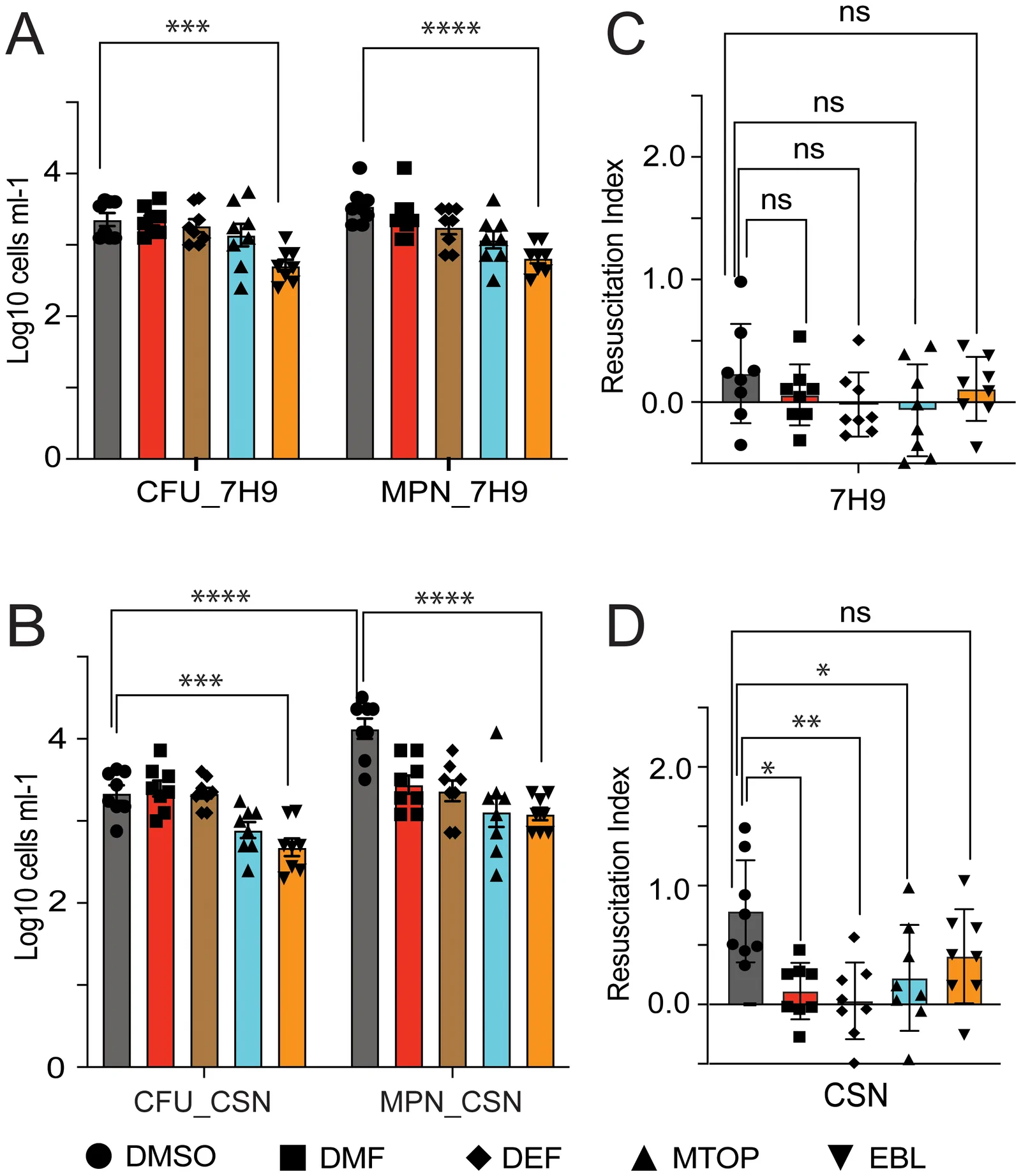
Effect of DMF and DMF derivatives on DC Mtb persisters generated in IFN-γ-activated macrophages. J774A.1 cell were preactivated with IFN-γ-(10 ng/mL) for 24 h following by infection with Mtb at MOI 1. Infected macrophages were then treated with compounds (25 μM) or DMSO for 24 h prior to determination of CFU and MPN counts. Results from two independent experiments performed in biological quadruplicates are shown as mean ± SEM (N = 8). CFU and MPN counts of Mtb are shown in panels **(A, B)**, **(A)** CFU_7H9 and MPN_7H9 counts; **(B)** CFU_CSN and MPN_CSN counts. Resuscitation indices (RIs) were calculated for 7H9 medium **(C)** and CSN **(D)** as RI_7H9 = Log_10_ (MPN_7H9 mL^-1^) − Log_10_ (CFU_7H9 mL^-1^) and RI_CSN = Log_10_ (MPN_CSN mL^-1^) − Log_10_ (CFU_CSN mL^-1^). *p<0.05, **p<0.01, ***p<0.001, ****p<0.0001; ns, non-significant (unpaired t-test or one way, ANOVA).

The NOD model was designed to evaluate the direct effects of DMF or its derivatives on DC Mtb, whereas the macrophage infection model captures both direct and indirect effect mediated by changes in the intracellular macrophage environment.

Exposure of logarithmic-phase Mtb cultures (log_10_CFUml^-1^ = 7) with NOD for 24 h, markedly reduced CFU counts, however, no statistically significant differences were observed between treatment groups (**Figure 6**). Recovery in 7H9 broth was lower than on 7H10 agar (**Figure 6A**), whereas growth was enhanced in culture supernatant-supplemented (CSN) medium (**Figure 6B**).

NOD treatment generated DC Mtb as indicated by a resuscitation index (RI) greater than 1, reflecting at least a ten-fold excess of bacteria recovered in CSN medium compared with colony-forming cells. DMF at 25 μM produced a modest non-significant reduction in RI, while DEF, MTOP, and EBL had no effect. These findings suggest that neither DMF nor its derivatives directly eliminate DC *Mtb* generated *in vitro*.

In the macrophage infection model, CFU and MPN counts in 7H9 medium (CFU_7H9 and MPN_7H9) and CFU and MPN counts in CSN medium (CFU_CSN and MPN_CSN) were determined (**Figure 7**). As reported previously (Gap-Gaupool et al., 2024), MPN_CSN counts in control (DMSO- treated) samples were significantly higher than CFU and MPN_7H9 counts (P< 0.01, one way ANOVA), confirming the presence of DC Mtb. EBL treatment resulted in significantly reduced CFU and MPN counts, consistent with its bactericidal activity. Although all compounds affected MPN_7H9 and MPN_CSN counts, RI values calculated using MPN_7H9 did not differ significantly between groups (**Figure 7B**).

In contrast, both DMF and DEF significantly reduced RI values calculated using MPN_CSN (**Figure 7D**). MTOP and EBL also moderately decreased RI, likely due in part to compound-associated toxicity to macrophages. Together, these results demonstrate that while DMF and DEF do not directly kill DC *Mtb*, they effectively eliminate DC *Mtb* within infected macrophages. This highlights the importance of host-directed, anti-inflammatory activity, particularly inhibition of NO production, for targeting DC *Mtb* persisters.

## Discussion

Within the infected host, Mtb may reside in multiple niches that vary in their permissiveness for Mtb growth (Sarathy and Dartois, 2020). This heterogeneity plays a critical role in driving the emergence of distinct Mtb phenotypes, many of which exhibit increased tolerance to antimicrobial treatment (Gold and Nathan, 2017).

DC Mtb represent one such subpopulation and are particularly challenging to treat. DC Mtb escape detection by standard laboratory diagnostics, yet accumulating evidence indicates that they are associated with unfavourable treatment outcomes (Peters et al., 2022) (Decker et al., 2025), and TB relapse in animal models (Hu et al., 2015). DC Mtb are generated during infection and induced by host *in vivo* environmental factors including inflammatory responses, NO and ROS (Gap-Gaupool et al., 2024) (Saito et al., 2021). Host metabolic reprogramming during infection, including a shift towards aerobic glycolytic pathways is supported by transcriptomic analyses of TB sputa where DC Mtb are highly prevalent (Lai et al., 2021).

Host-directed therapy has therefore been proposed as a priority strategy for the development of novel TB interventions (Nathan and Barry, 2015) (Wong et al., 2026). Several non-steroidal anti-inflammatory drugs (Gold et al., 2012) and other immunomodulators have shown promising results against Mtb (Daniel and Bhakta, 2022). We have demonstrated that dimethyl fumarate (DMF), a well-established anti-inflammatory drug eliminates DC Mtb from infected mice (Glenn et al., 2022) and macrophages (Gap-Gaupool et al., 2024). Although DMF is approved for treatment of multiple sclerosis and psoriasis (Kourakis et al., 2020), it is associated with side effects, including reduced lymphocyte counts (Diebold et al., 2022) and potential interference with interferon gamma release assays (Dantas et al., 2021). Therefore, we hypothesised that DMF could be modified to improve its solubility and stability while reducing adverse effects.

In this study, we focussed on commercially available DMF-related compounds (**Table 1**) and established a simple workflow for the initial screening of candidates with anti-DC Mtb persister activity. Our results show that replacement of both methyl groups with ethyl moieties did not reduce compound potency against Mtb as evident from the MIC values for DMF and DEF, however, removal of one of the groups increased MICs eightfold in case of MMF or MEF (**Table 1**). Thus, both ester groups are required for optimal anti-Mtb activity.

Two additional compounds, MTOP and EBL, demonstrated higher potency against Mtb (MIC = 3.13 µM), and were active against both smooth and rough morphotypes of Mabs (MIC = 50 µM). Both compounds had bactericidal effect against Mtb and smooth morphotype of Mabs. Moreover, EBL, which is more lipophilic than other tested compounds, exhibited rapid bactericidal activity in a macrophage infection model (**Figure 7A**), identifying it as a promising candidate for further drug development. Mabs is notoriously resistant to many antibiotics, including TB drugs such as rifampicin and quinolones (Victoria et al., 2021). The identification of new bactericidal agents that interfere with Mabs metabolism is therefore of substantial clinical importance. Further increasing the potency of EBL and MTOP while reducing their cytotoxicity represent a plausible future direction. Other approved anti-inflammatory drugs, including ibuprofen, diclofenac and dexamethasone, exhibited high MICs against Mtb (≥400 µM) and were completely inactive against Mabs. Based on selectivity indices above 5, DMF, DEF, MTOP and EBL were selected for further testing.

Testing of NO production in LPS-stimulated macrophages, revealed an inhibitory effect by all compounds at a concentration of 25 µM. However, only DMF and DEF caused a reduction of nitrite concentrations above 80%. Aminoguanidine, a known iNOS inhibitor, completely abolished NO production in all conditions tested (**Figure 5**).

DMF exerts complex pleiotropic effects on eukaryotic cells, including activation of antioxidant responses, suppression of inflammatory pathways, and metabolic reprogramming (Kourakis et al., 2020). Identified targets of DMF include nuclear factor erythroid 2-related factor (Nrf2) (Bruck et al., 2018), nuclear factor kappa-B (NF-κB) (Kourakis et al., 2020), the glycolytic enzyme glyceraldehyde 3-phosphate dehydrogenase (GAPDH) (Kornberg et al., 2018), NADPH oxidase 3 (a major source of ROS) and gasdermin D (Humphries et al., 2020). Despite these insights, the precise mechanism of action of DMF remains incompletely understood. DMF modifies cysteine residues through the formation of *S*-(2-succinyl)-cysteine, leading to inactivation of its targets both in eukaryotic cells (Humphries et al., 2020) and mycobacteria (Ruecker et al., 2017).

The mechanism by which DMF eliminates DC Mtb persister in infected mice and macrophages remains unclear. To address this, we investigated whether DMF or related compounds directly affected DC Mtb generated *in vitro* in the absence of host-mediated pathways. As shown in **Figure 6**, none of the tested compounds had a statistically significant effect on DC Mtb formed after exposure to NOD. In contrast, all compounds reduced Mtb resuscitation from IFN-γ treated macrophages. However, only DMF and DEF significantly reduced RI_CSN_ values.

These findings have important implications for drug development. DEF is more soluble in DMSO and more potent against Mtb than is DMF (**Table 1**) and showed comparable anti-persister activity (**Figure 7**). MTOP and EBL exhibit bactericidal activity against Mtb and Mabs and their modest anti-persister effects may be enhanced through rational medicinal chemistry optimisation.

This study has several limitations. First, for testing anti-DC Mtb persister effect we used a murine macrophage cell line that produces high levels of NO following LPS or IFN-γ stimulation. Although our findings are consistent with those from a murine infection model and suggest that cell lines may be suitable for initial validation, further studies in primary human macrophages are required. The role of NO in human TB remains controversial (Kong et al, 2025), with evidence indicating variable levels of NO production in humans, effects on Mtb physiology (Tarekegn et al., 2024) and limited NO production by human macrophages (Jamaati et al., 2017). Consequently, Mtb persisters may respond differently to DMF and its derivatives in human macrophages. We are currently developing primary human macrophage models to assess the activity of compounds against DC Mtb persisters.

Second, a more comprehensive assessment of the proinflammatory response is needed to understand the mechanisms underlying DCMtb formation and persistence. This would also facilitate the development of models with controlled cytokine responses for evaluating drug targeting DC Mtb persisters.

Future validation in animal infection models and primary human macrophages, together with additional optimisation of DMF and DEF, will be important to strengthen the findings of this study and support the development of more potent compounds. Overall, our findings suggest that anti-DC Mtb persister activity of DMF and DEF may be mediated through modulation of the host immune response rather than solely through direct action on Mtb. Importantly, their anti-inflammatory effects appear to be complemented by intrinsic antimycobacterial activity, raising the possibility that other anti-inflammatory drugs may also have therapeutic potential against DC Mtb. Given the critical role of host metabolic reprogramming during infection, targeting host metabolism represents a promising area for future therapeutic intervention.

## Conclusion

We developed a cost-effective and straightforward workflow for the initial assessment of compounds with anti-DC Mtb persister activity. This workflow can be adapted for high-throughput screening and applied to the evaluation of drugs against other heterogeneous Mtb populations. Using this approach, we identified diethyl fumarate (DEF) as a promising compound with dual antimycobacterial and anti-inflammatory activity that warrants further optimisation for the development of anti-DC Mtb persister therapies. These findings provide a foundation for the development of host-directed therapeutic strategies aimed at accelerating bacterial clearance and reducing treatment duration.

## Data Availability

The raw data supporting the conclusions of this article will be made available by the authors, without undue reservation.
